# Supplementary material for: Access to Information and Communication Technology, Digital Skills, and Perceived Well-Being among Older Adults in Hong Kong
Source: Int J Environ Res Public Health. 2023 Jun 23;20(13):6208. doi: 10.3390/ijerph20136208 (PMC10340767; doi:10.3390/ijerph20136208)
Supplement: Supplementary file 1 [file ijerph-20-06208-s001.zip › ijerph-2334982-supplementary.pdf]

International Journal of
Environmental Research and Public Health

MDPI

Tables of data analysis

Table S1. Sample characteristics and gender differences in major variables (N = 1018)

	Total		Male (n = 358)		Female (n = 660)			
Variables	M	SD	M	SD	M	SD	t	p
Age [a]	72.86	8.74	72.46	8.84	73.10	8.69	−1.010	0.313
Numbers of devices	1.54	1.20	1.85	1.30	1.38	1.11	6.102	<0.001
Variables	%		%		%		χ2 (df)	p
Education level							52.977 (7)	<0.001
Never attend	7.6%		4.2%		9.4%			
Primary school	25.9%		17.3%		30.6%			
Secondary school	17.8%		17.9%		17.7%			
Associate degree	24.1%		27.4%		22.3%			
Bachelor’s degree	7.7%		12.8%		4.8%			
Master’s degree or above	8.9%		11.7%		7.4%			
Self-perceived class status							12.178 (5)	0.032
Upper class	0.7%		1.6%		.2%			
Upper middle class	3.5%		3.4%		3.5%			
Middle class	26.9%		27.1%		26.8%			
Lower middle class	20.8%		24.6%		18.7%			
Grassroot	34.4%		31.5%		35.9%			
Refuse to answer	13.8%		11.8%		14.8%			

Notes

[a] N = 305 and 517, respectively, for male and female participants; [b] N = 257 and 391, respectively, for male and female participants

Table S2. Material access to ICT, frequency of access and gender differences

	Total		Male (n = 358)		Female (n = 660)			
**Variables**	M	SD	M	SD	M	SD	t	p
**Internet usage** [b]	11.37	2.21	10.98	2.17	11.62	2.21	−3.636	<0.001
**Digital skills** [b]	81.03	28.28	86.90	27.06	77.17	28.44	4.345	<0.001
**Variables**	%		%		%		χ2 (df)	p
**Living alone**	24.7%		21.8%		26.2%		2.850 (2)	0.240
**Digital device**								
**Desktop**	24.4%		13.2%		11.2%		51.18(1)	0.000
**Laptop**	23.6%		11.4%		12.2%		23.877(1)	0.000
**Mobile phone**	78.2%		28.7%		49.5%		3.681(1)	0.055
**Tablet**	28.1%		11.8%		16.3%		8.045(1)	0.005
**None of these**	18.2%		5.3%		12.9%		3.133(1)	0.077
**Internet access at home or mobile phone data plan**							26.866 (2)	<0.001
**None**	23.5%		19.0%		25.9%			
**Either**	23.2%		16.8%		26.7%			
**Both**	53.3%		64.2%		47.4%			
**Frequency of Internet access**							22.525 (5)	<0.001
**Never**	36.3%		28.2%		40.8%			
**Less than once a month**	1.8%		1.1%		2.1%			
**Once a month**	1.6%		1.4%		1.7%			
**Once a week**	4.0%		3.1%		4.5%			
**Every day**	8.6%		10.6%		7.6%			
**Several times per day**	47.6%		55.6%		43.3%			
**Any purely online friendship**	6.1%		8.9%		4.5%		7.832 (1)	0.005

Notes

[a] N = 305 and 517, respectively, for male and female participants

[b] N = 257 and 391, respectively, for male and female participants

Table S3. Frequency of perceived easiness of digital usage (response = easy or very easy) and gender differences

Perceived easiness	Total (N = 648)	Male (N = 257)	Female (N = 391)
Q11. Adjust the screen brightness.	76.40%	83.30%	71.9% ***
Q10. Navigate onscreen menus using the touchscreen	75.60%	76.30%	75.20%
Q25. Read the news on the Internet.	69.30%	76.70%	64.5% ***
Q21. Use search engines (e.g. Google, Bing).	68.50%	74.30%	64.70%
Q23. Find information about my hobbies and interests on the Internet.	67.40%	74.30%	62.90%
Q12. Connect to a Wi-Fi network.	66.40%	77.00%	59.30% ***
Q35. Close games and other applications.	66.00%	66.90%	65.50%
Q32. Watch movies and videos.	65.00%	64.60%	65.20%
Q24. Find health information on the Internet.	64.50%	67.70%	62.40%
Q36. Delete games and other applications.	63.40%	69.30%	59.60%
Q13. Open emails.	59.00%	66.50%	54.0% ***
Q22. Find information about local community resources on the Internet.	53.10%	59.90%	48.60%
Q16. View pictures sent by email.	51.20%	60.30%	45.3% ***
Q27. Save text and images I find on the internet.	50.80%	56.80%	46.80%
Q34. Update games and other applications.	47.80%	52.90%	44.50%
Q37. Upgrade device sofiware.	47.20%	56.40%	41.2% ***
Q14. Send emails.	47.10%	53.70%	42.70%
Q29. Check the date and time of upcoming and prior appointments.	44.80%	47.90%	42.70%
Q28. Enter events and appointments into a calendar.	43.10%	47.10%	40.40%
Q17. Send pictures by email.	41.00%	47.50%	36.80%
Q31. Use the devices online store to find games and other forms of entertainment (e.g. using Apple App Store or Google Play Store).	40.30%	43.20%	38.40%
Q30. Set up alerts to remind me of events and appointments.	37.50%	42.00%	34.50%

Q15. Store email addresses in an email address book or contact list.	37.30%	46.30%	31.5% ***
Q26. Bookmark websites to find them again later (make favorites).	34.10%	41.20%	29.40%
Q33. Read a book.	29.00%	32.30%	26.90%
Q18.Transfer information (files such as music, pictures, documents) on my mobile device to my computer.	28.20%	35.40%	23.5% ***
Q20. Store information with a service that lets me View my files from anywhere (e.g. Dropbox, Google Drive, Microsoft Onedrive).	24.20%	26.50%	22.80%
Q19. Transfer information (files such as music, pictures, documents) on my computer to my mobile device.	23.90%	30.70%	19.4% ***

Notes

*** As the Bonferroni correction was applied to correct for multiple chi-square tests (df = 1), the significance level was set at .0013

Table S4. Differences between participants who never or rarely used Internet and those who used Internet every day

	a) Never used Internet (n = 370)	b) Less than once a month to once a week (n = 75)	c) Every day (n = 573)		
Variables	% / M (SD)	% / M (SD)	% / M (SD)	F (post-hoc) / χ2 (df)	p
Age	78.35 (8.80)	71.45 (6.51)	69.37 (6.89)	128.193 (b>a, c>a)	<0.001
Gender (female)	72.7%	73.3%	58.6%	22.070 (2)	<0.001
Education level				200.335 (12)	<0.001
Never attend	16.2%	10.0%	2.7%		
Primary school	42.2%	40.0%	16.6%		
Secondary school	20.6%	18.6%	17.5%		
Associate degree	14.5%	15.7%	33.1%		
Bachelor's degree	4.4%	8.6%	10.2%		
Master's degree or above	1.5%	4.3%	14.8%		
Self-perceived social class				71.000 (10)	<0.001
Upper class	0.6%	0.0%	0.8%		
Upper middle class	1.5%	1.5%	5.0%		
Middle class	17.5%	23.9%	33.4%		
Lower middle class	17.2%	14.9%	23.9%		
Grassroot	42.8%	37.3%	28.5%		
Refuse to answer	20.5%	22.4%	8.3%		
Satisfied with current life in the past week	58.4%	70.7%	76.6%	42.928 (4)	<0.001
Feeling empty in daily life in the past week	20.5%	17.3%	14.8%	33.325 (4)	<0.001
Often feeling happy in the past week	44.6%	46.7%	54.5%	9.277 (4)	0.055
Self-rated physical health	3.11 (1.01)	3.23 (0.88)	3.49 (8.46)	20.254 (c>a)	<0.001
Self-rated mental health	3.42 (0.95)	3.45 (0.93)	3.79 (0.82)	21.429 (c>a, c>b)	<0.001

Table S5. Hierarchical multiple regression predicting self-rated health (SRH) and self-rated mental health (SRMH) (N = 507)

	SRH				SRMH			
	Model 1		Model 2		Model 1		Model 2	
Variables	β	p	β	p	β	p	β	p
Constant		0.000		0.000		0.000		0.000
Gender (male)	−0.005	0.915	0.006	0.902	0.026	0.573	0.050	0.288
Education level	0.004	0.940	−0.006	0.919	0.000	0.994	−0.044	0.408
Self-perceived social class	−0.188	0.000	−0.176	0.001	−0.274	0.000	−0.243	0.000
Age	−0.020	0.675	−0.017	0.739	−0.045	0.326	−0.011	0.824
Digital skills			0.159	0.044			0.232	0.003
Internet usage			0.132	0.085			0.102	0.172
R^2	0.037		0.046		0.079		0.100	
Adjusted R^2	0.028		0.033		0.071		0.088	
F	4.294 **		3.597 **		9.633 ***		8.231 ***	
ΔR^2	0.037		0.009		0.079		0.021	
ΔF	4.294 **		2.160		9.633 ***		5.075 **	

** *p* < .01 *** *p* < .001
